# Postharvest chitosan-arginine nanoparticles application ameliorates chilling injury in plum fruit during cold storage by enhancing ROS scavenging system activity

**DOI:** 10.1186/s12870-022-03952-8

**Published:** 2022-12-02

**Authors:** Roghayeh Mahmoudi, Farhang Razavi, Vali Rabiei, Lluís Palou, Gholamreza Gohari

**Affiliations:** 1 Department of Horticulture, Faculty of Agriculture, University of Zanjan, Zanjan, Iran; 2 Postharvest Technology Center (CTP), Valencian Institute of Agrarian Research (IVIA), 46113 Montcada, Valencia Spain; 3 Department of Horticulture, Faculty of Agriculture, University of Maragheh, Maragheh, Iran

**Keywords:** Amino acid, Edible coating, Nanotechnology, *Prunus domestica*, Shelf life

## Abstract

**Background:**

Plum (*Prunus domestica* L.) has a short shelf-life period due to its high respiration rate and is sensitive to low storage temperatures, which can lead to the appearance of chilling injury symptoms. In this investigation, we applied new coating treatments based on chitosan (CTS) and arginine (Arg) to plum fruit (cv. ‘Stanley’).

**Results:**

Fruit were treated with distilled water (control), Arg at 0.25 and 0.5 mM, CTS at 1% (w/v) or Arg-coated CTS nanoparticles (CTS-Arg NPs) at 0.5 and 1% (w/v), and then stored at 1 °C for days. The application of CTS-Arg NPs at 0.5% attenuated chilling injury, which was accompanied by accumulation of proline, reduced levels of electrolyte leakage and malondialdehyde, as well as suppressed the activity of polyphenol oxidase. Plums coated with CTS-Arg NPs (0.5%) showed higher accumulation of phenols, flavonoids and anthocyanins, due to the higher activity of phenylalanine ammonia-lyase, which in turn resulted in higher DPPH scavenging capacity. In addition, CTS-Arg NPs (0.5%) treatment delayed plum weight loss and retained fruit firmness and ascorbic acid content in comparison to control fruit. Furthermore, plums treated with CTS-Arg NPs exhibited lower H_2_O_2_ accumulation than control fruit due to higher activity of antioxidant enzymes, including CAT, POD, APX and SOD.

**Conclusions:**

The present findings show that CTS-Arg NPs (0.5%) were the most effective treatment in delaying chilling injury and prolonging the shelf life of plum fruit.

## Background

Low-temperature storage is the most important postharvest strategy to preserve horticultural crops, contributing to extend the shelf life and maintain the nutraceutical quality. Low-temperature storage not only reduces respiration rate but also lessens physiological disorders during storage [1]. However, the high sensitivity of plums to low-temperature storage can lead to chilling injury, which causes substantial alterations in the fruit appearance, such as flesh browning, flesh bleeding and flesh translucency [2]. In the last years, diverse treatments, such as modified atmosphere packaging, 1-methylcyclopropane [3], edible coatings [4, 5], nitric oxide fumigation [6], melatonin [7, 8] and chlorine dioxide [9], have been employed for reducing chilling injury and extending the postharvest life of plums. Among these approaches, edible chitosan coatings have been widely used due to their biocompatibility, biodegradability and non-toxicity [10]. Chitosan (CTS), the second most abundant natural polysaccharide in nature after cellulose, is generally obtained from deacetylation of chitin extracted from the exoskeletons of crustaceans [11]. CTS has been effective for many applications in the field of agriculture, such as ameliorating crop yield, promoting plant growth and controlling diseases and physiological disorders in fruits and vegetables [12]. Different studies showed that CTS coating is an effective method to maintain postharvest quality, prolong shelf-life and increase antioxidant activity in fresh fruits such as sweet cherry [13], plum [14], Japanese plum [5, 15] and strawberry [16].

Arginine (Arg), a metabolically active amino acid found in the structural units of many important proteins, has exhibited activity in enhancing the tolerance against chilling stress in horticultural crops. Arg has physiological roles in living cells as a precursor for the biosynthesis of polyamines (PAs), proline, γ-aminobutyric acid (GABA) and signaling molecules such as nitric oxide (NO) [17, 18]. In several studies, Arg inhibited the senescence of strawberry [19], green asparagus [20] and button mushroom [21]. Sohail et al. [22] showed that Arg prevented the senescence of broccoli by maintaining the green color and reducing the levels of ethylene production and respiration. In recent years, researchers have become interested in the impact of Arg on environmental stress responses. Pretreatment of sunflower seeds with Arg increased resistance to drought stress by enhancing the accumulation of polyamines [23]. Babalar et al. [24] stated that postharvest Arg application to pomegranate fruit during cold storage alleviated chilling injury by retaining the membrane cell integrity, increasing the scavenging activity of reactive oxygen species (ROS) and lowering H_2_O_2_ accumulation.

Nanotechnology focuses typically on the study and application of nanoparticles (NPs) in the range of 1–100 nm. NPs are extensively used in the horticultural sector, including for the control of postharvest physiological disorders and diseases of fruits and vegetables [25]. NPs exhibit impressive mobility in plants due to different intrinsic characteristics, such as small size, structural variety and high surface to volume ratio, which are associated with increased catalytic activity and changes in plant metabolism [26]. Xing et al. [27] stated that the application of a chitosan/nano TiO_2_ composite coating retained nutritional quality and extended shelf life of blueberry fruit for up to 32 days of storage. Song et al. [28] showed that a chitosan/nano-silica coating augmented chilling tolerance of loquat fruit by increasing the activity of antioxidant enzymes and decreasing sugars content. In general, the incorporation of NPs into coating formulations for fresh produce can play a crucial role in maintaining the postharvest quality, increasing ROS scavenging activity and extending the shelf life period. In this work, chitosan-Arginine nanoparticles (CTS-Arg NPs) were synthesized and applied to harvested plum fruit to investigate their potential effects on physiological disorders, enzymatic and antioxidant activity, postharvest fruit quality and storability at low temperature.

## Results and discussion

### Characterization of CTS-Arg NPs

**Fig. 1:**
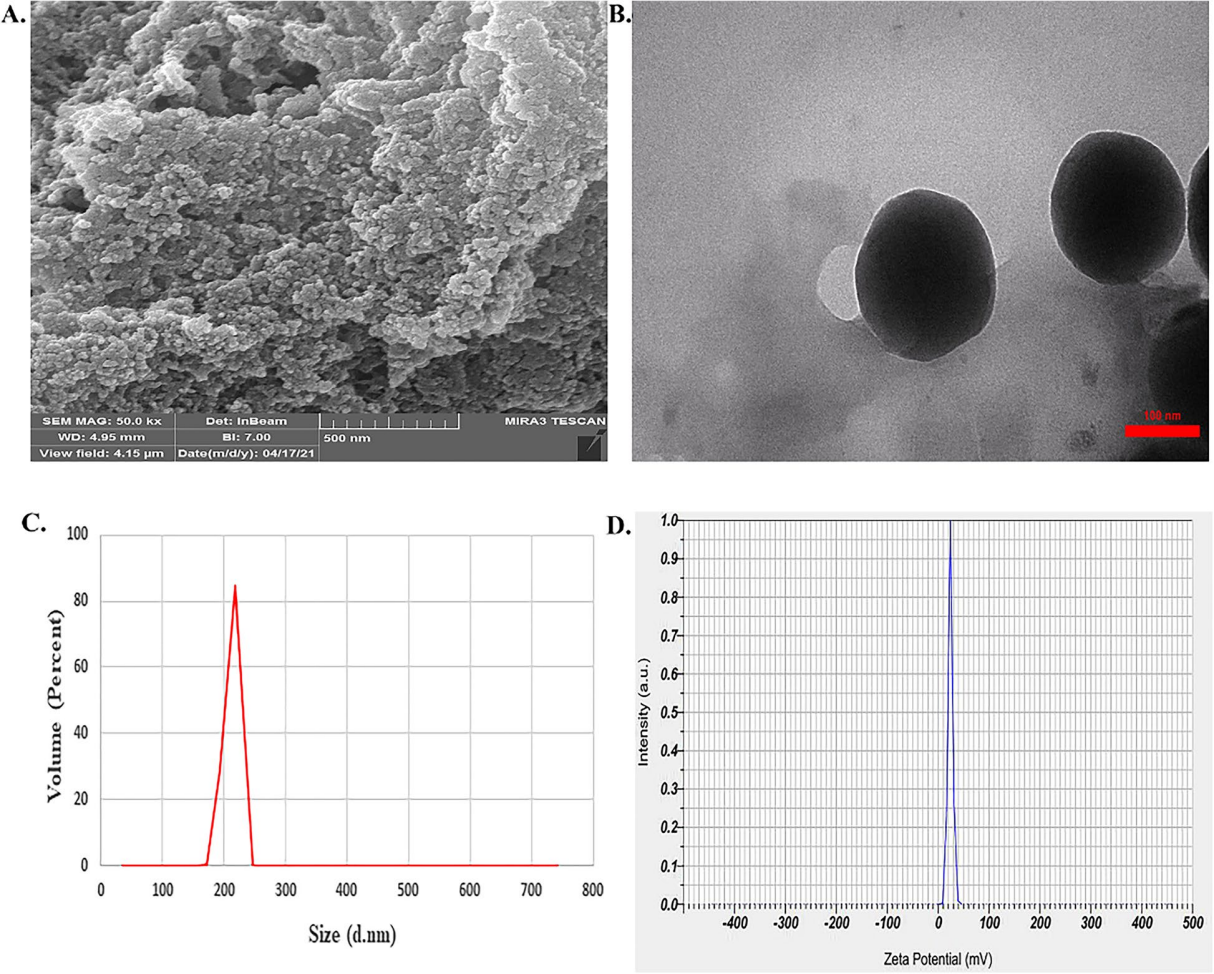
SEM (**A**), TEM (**B**), DLS (**C**), and zeta potential (**D**) result of chitosan-arginine nanoparticles (CTS-Arg NPs)

The freeze-dried nanocomposite powder was characterized by scanning electron microscope (SEM) and transmittance electron microscopy (TEM) techniques. Figure 1A shows the SEM image of nanocomposite composed of spherical nanoparticles. In Fig. 1B, the TEM image is illustrated spherical shape of nanoparticles with the diameter size of ~ 270 nm. The particle size was analyzed using the Dynamic Light Scattering (DLS) technique. The size of CTS-Arg NPs is shown in Fig. 1C. The average particle diameter and polydispersity index (PDI) value were 218 nm and 0.842 nm, respectively. In addition, the results of zeta potential are shown in Fig. 1D. This value was obtained for CTS-Arg NPs + 24 mV.

### Chilling injury and membrane integrity

**Fig. 2:**
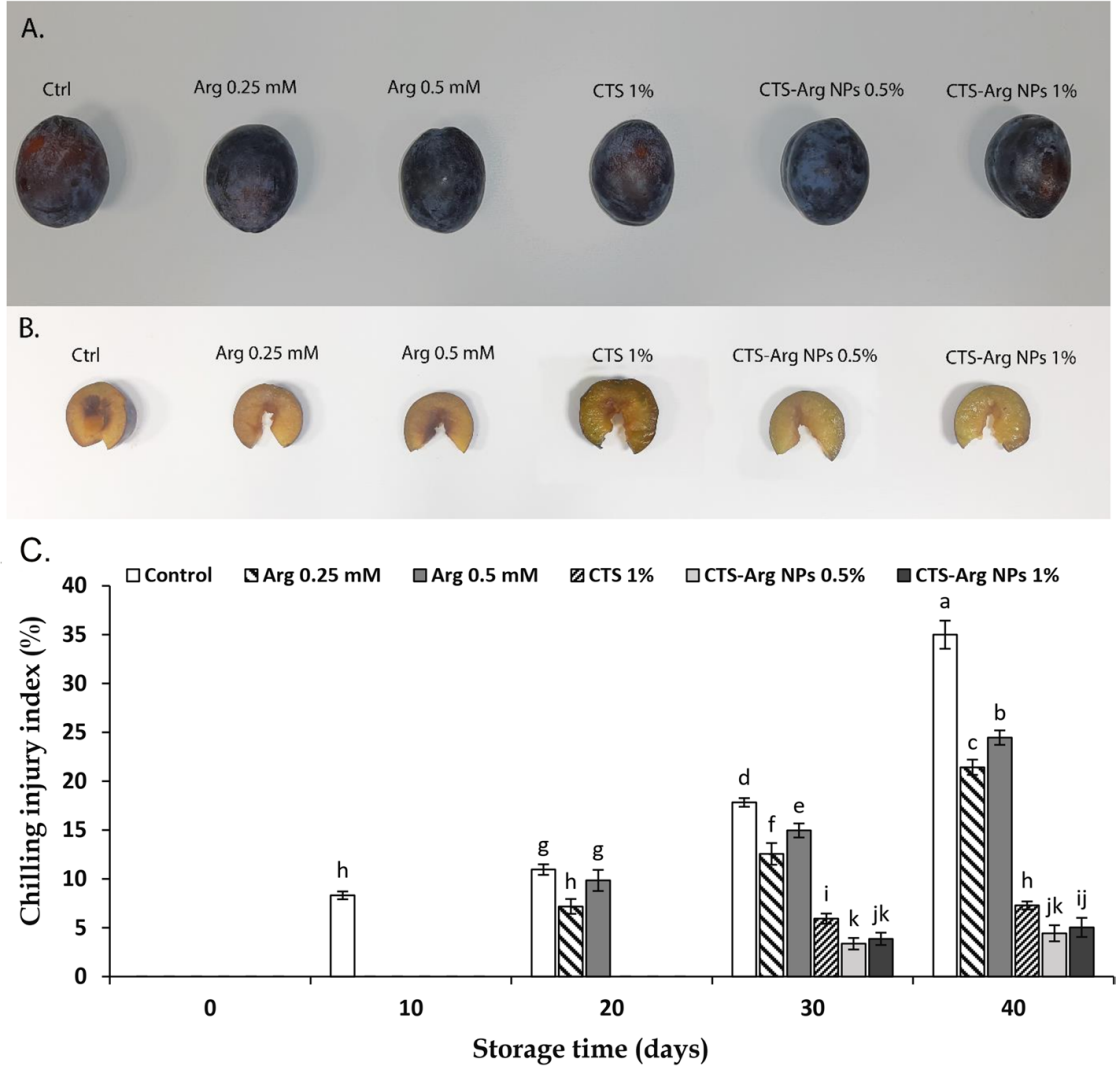
The Effect of control (no treatment), arginine (Arg), chitosan (CTS) and CTS-Arg nanoparticles (NPs) treatments on fruit shape (**A**) and pulp browning (**B**) and chilling injury index (**C**) in ‘Stanley’ plums in 40-day storage at 1 °C

In the current study, plum fruit showed flesh browning and translucency as main symptoms of chilling injury at 1 °C during 40 days of storage (Fig. 2A, B).

Irrespective of the treatments, as well as temperature of storage, chilling injury remarkably enhanced in plum fruits during the whole cold storage. Fruit coated with CTS-Arg NPs (0.5 and 1%) exhibited the lowest rate (4.43 and 5.03%, respectively) of chilling injury in comparison to Arg and CTS individual treatments (Fig. 2C). Also, there were no differences between concentrations of 0.5 and 1% CTS-Arg NPs.

**Fig. 3:**
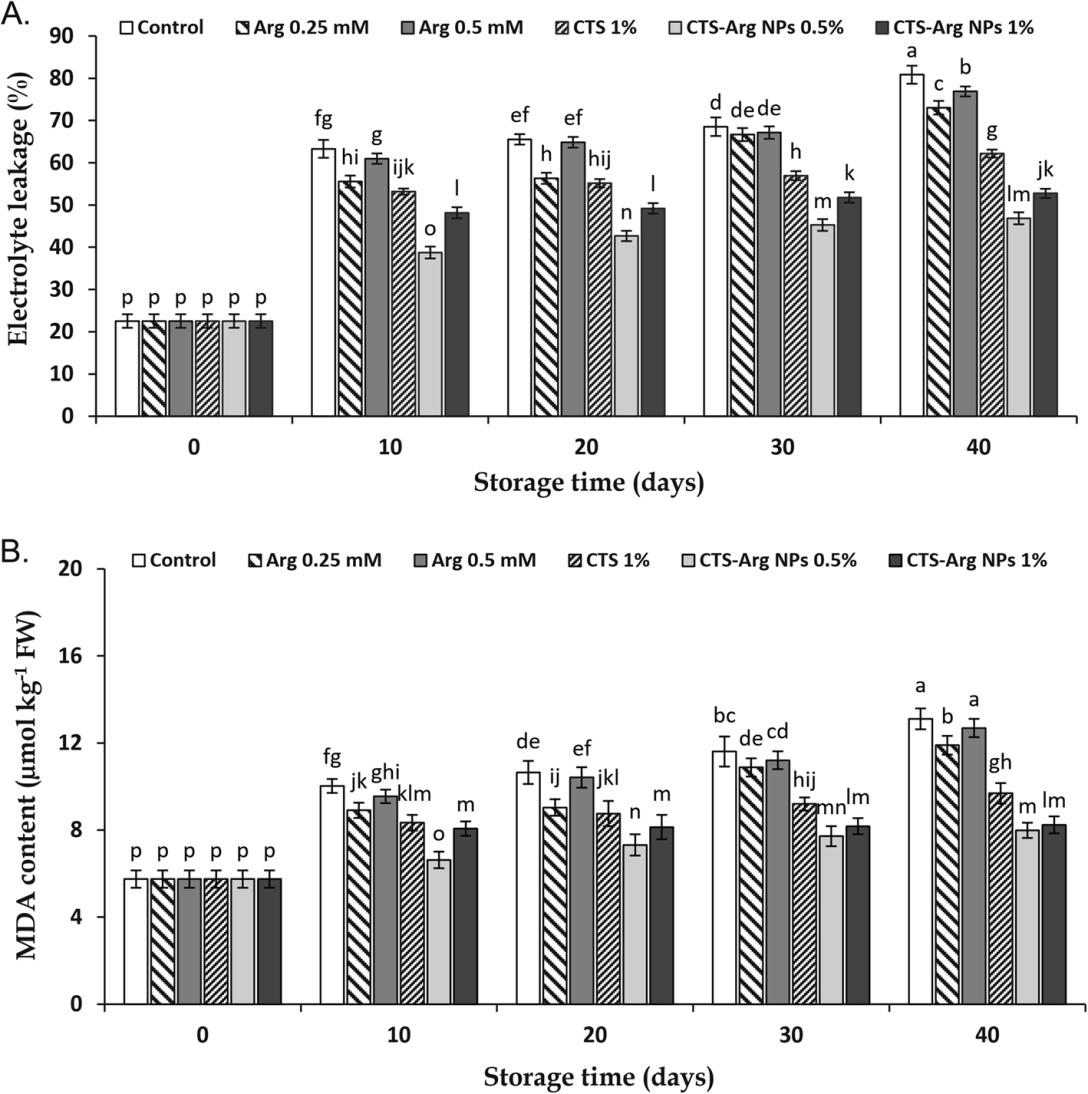
Effect of control (no treatment), arginine (Arg), chitosan (CTS) and CTS-Arg nanoparticles (NPs) treatments electrolyte leakage (**A**) and MDA accumulation (**B**) in ‘Stanley’ plums during storage at 1 °C for 40 days. Values shown are means ± standard errors (*n* = 3). Different letters above the bars indicate significant differences at *P* < 0.05 according to Duncan’s test

Being coupled with increases of electrolyte leakage and MDA content, chilling injury symptoms appearance and damage of cell membranes were recorded. According to Fig. 3A, B, electrolyte leakage and MDA content at harvest time were 22.53% and 5.75 μmol kg^− 1^ FW, respectively, which increased in all samples during storage. At the end of the storage period, 0.25 and 0.5 mM Arg-treated plums and control showed higher levels of EL, whereas the application of CTS-Arg NPs (0.5%) delayed EL increase (Fig. 3A). Seemingly, fruits treated with 0.5% CTS-Arg NPs showed the lowest electrolyte leakage (46.84%) after 40 days of storage as compared to control (80.83%) and maintained the highest membranes integrity in contrast to other treated fruits. Increments of MDA content were observed in all untreated and treated plums (Fig. 3B). The highest MDA content was observed in control (13.11 μmol kg^− 1^ FW) and 0.5 mM Arg (12.69 μmol kg^− 1^ FW). Compared to control plums, CTS-Arg NPs coatings (0.5 and 1%) remarkably inhibited or buffered the accumulation of MDA (7.99 and 8.24 μmol kg^− 1^ FW, respectively) at the end of the 40-day storage period, although there were no differences among these treatments (Fig. 3B).

The susceptibility of plum fruit to low temperatures may lead to the appearance of chilling damage symptoms [2, 29]. In the present study, control fruit displayed severe chilling symptoms whereas they decreased substantially in fruit coated with CTS-Arg NPs. Zhang et al. [30] found that exogenous 0.2 mM Arg treatment reduced CI index in comparison with control tomato fruit. Babalar et al. [24] reported that the postharvest application of Arg reduced chilling injury in pomegranate fruit by decreasing EL. Nasr et al. [31] expressed that chitosan-phenylalanine nanoparticles (Cs-Phe NPs) increased persimmon tolerance to chilling injury by maintaining the cell membrane integrity and reducing the levels of reactive oxygen species (ROS). Peroxidation of the cell membrane arising from cold stress and the accumulation of ROS leads to damage the membrane integrity, which can be evaluated by measuring EL and MDA accumulation [32]. The current findings showed that EL and MDA content in treated and control fruit showed an ascending trend during cold storage, but the application of the coating CTS-Arg NPs effectively suppressed the increment of EL and the accumulation of MDA. Similarly, Sohail et al. [22] and Shu et al. [19] reported that EL and MDA content in samples treated with Arg were remarkably lower than in control samples. Moreover, the use of a chitosan / Nano TiO_2_ coating induced a lower accumulation of MDA in blueberries than the use of CTS alone [27]. In general, it seems that CTS-Arg NPs (0.5%) treatment delayed chilling injury by reducing oxidative stress, maintaining the membrane integrity and controlling EL and the accumulation of MDA.

### Fruit quality parameters

**Fig. 4:**
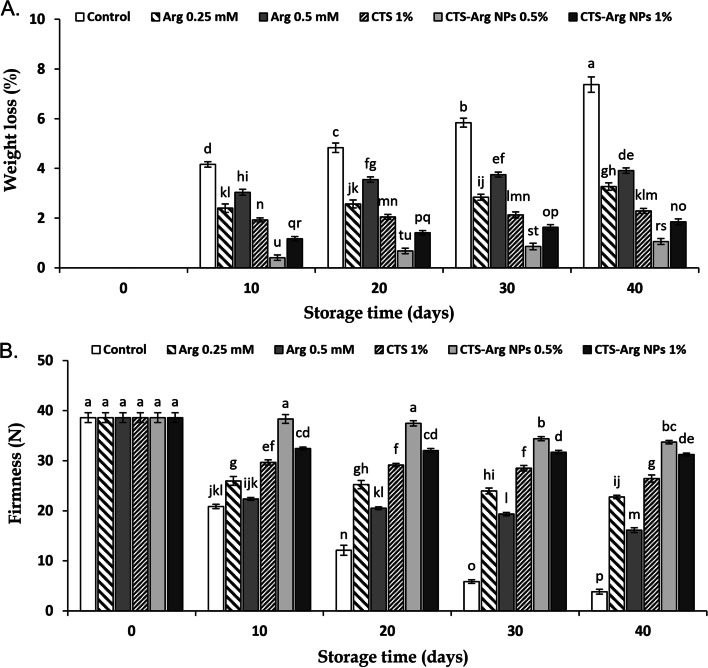
Effect of control (no treatment), arginine (Arg), chitosan (CTS) and CTS-Arg nanoparticles (NPs) treatments on weight loss (**A**) and firmness (**B**) in ‘Stanley’ plums during storage at 1 °C for 40 days. Values shown are means ± standard errors (*n* = 3). Different letters above the bars indicate significant differences at *P* < 0.05 according to Duncan’s test

Weight loss of all treated and control plums showed an upward trend during days of cold storage. All treated fruit retained weight loss in comparison with control fruit, and CTS-Arg NPs at 0.5% was the most effective treatment. At the last sampling time, weight loss in control samples was 7.37% and in plums treated with 0.5 and 1% CTS-Arg NPs 1.06 and 1.85%, respectively (Fig. 4A). The results exhibited that fruit firmness at harvest time was 38.6 N and gradually reduced in all treatments during 40 days of storage at 1 °C (Fig. 4B). The firmness of fruit tissue in all treated plums decreased during storage, but in a lower proportion than in control fruit. The lowest firmness level was measured in control and 0.5 mM Arg (3.83 and 16.13 N), respectively. However, CTS-Arg NPs treatments maintained the higher firmness rate than other treatments at the end of storage. Seemingly, plums coated with CTS-Arg NPs at 0.5% exhibited the lowest softening rate in the interval of 10 to 40 days of cold storage.

The main reason accounting for fruit weight loss is water loss produced by increasing transpiration and respiration rates after harvest [33]. The extension of storage time and destruction of cell wall compounds are the main reasons accounting for the increasing softening of fruit tissues [34]. Hassan et al. [35] stated that 0.5 mM L-arginine concentration enhanced fruit firmness and prevented weight loss of cucumber fruits during the whole storage period. Higher integrity of membrane in arginine-treated pomegranate deferred the softening and preserved higher fruit firmness through polyamines accumulation [24]. Meena et al. [36] reported that tomatoes treated with Cu-chitosan NPs maintained fruit firmness during 12 days of storage. According to our results, plums coated with CTS-Arg NPs (0.5%) controlled weight loss and retarded softening of fruit tissue.

### Proline content

**Fig. 5:**
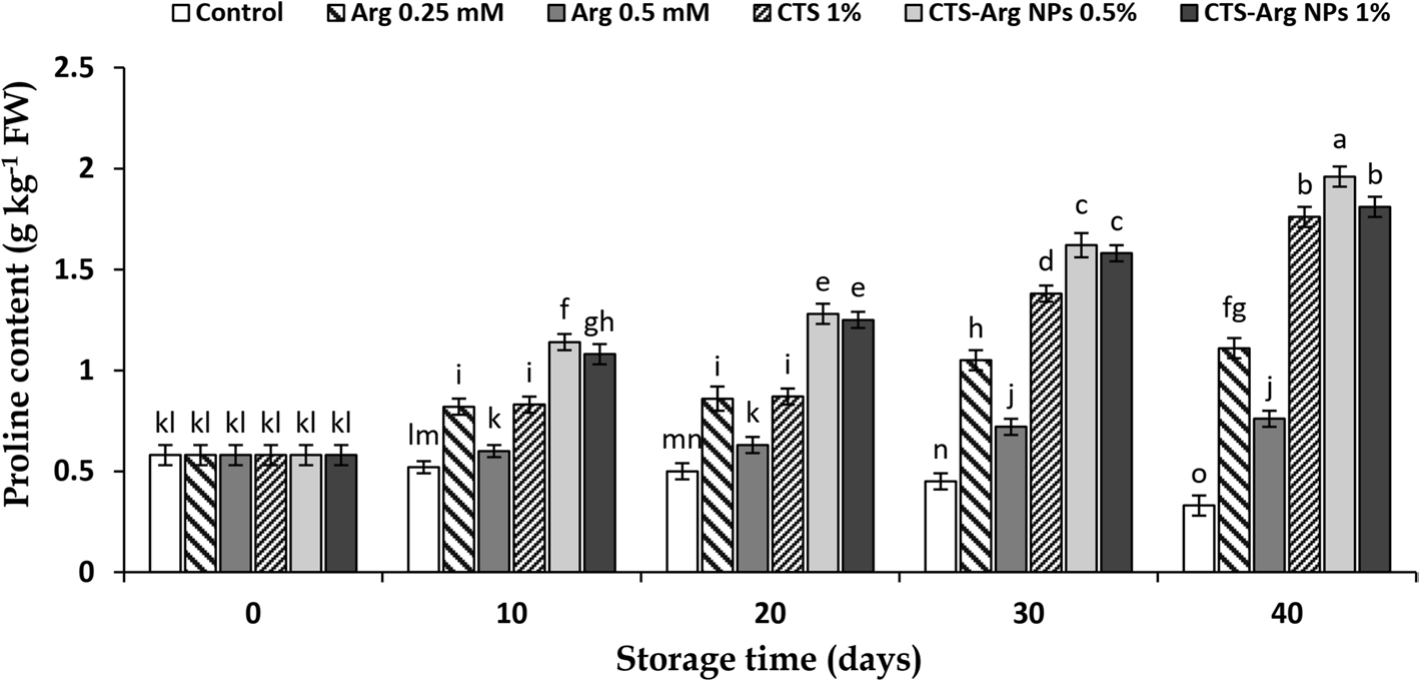
Effect of control (no treatment), arginine (Arg), chitosan (CTS) and CTS-Arg nanoparticles (NPs) treatments on proline content in ‘Stanley’ plums during storage at 1 °C for 40 days. Values shown are means ± standard errors (*n* = 3). Different letters above the bars indicate significant differences at *P* < 0.05 according to Duncan’s test

As shown in Fig. 5, the content of fruit proline at harvest time was 0.58 g kg^− 1^ FW. The proline content increased in all treated plum samples during the entire storage period. The highest content of proline was seen at the end of storage. After 40 days of storage, the highest (1.96 g kg^− 1^ FW) and lowest (0.33 g kg^− 1^ FW) values of proline were registered in plums treated with CTS-Arg NPs (0.5%) and in control plums, respectively. On the other hand, no difference was found between CTS-Arg NPs (1%) and CTS (1%) treatments at the last sampling time (Fig. 5).

The amino acid proline has main functions in higher plants under stress conditions: (1) ROS and OH^−^ radicals scavenging, (2) consolidation of cell membrane structure and (3) the accumulation of energy for metabolism [17, 37]. Zhang et al. [38] noticed that in cherry tomato fruits, Arg treatment enhanced proline content with higher arginase activity, resulting in lower chilling injury during storage at 2 °C. Our results were consistent with the findings of Zhang et al. [38] who reported that proline content increased in tomatoes treated with 0.2 mM Arg along with recovery of chilling injury under chilling stress. Furthermore, the foliar application of Cs-Se NPs remarkably increased proline content in bitter melon under salt stress [39]. Mahmoudi et al. [40] reported that CTS-GB NPs treatment (0.5%) enhanced proline content in plum fruit during storage at 1 °C for 40 days. Accordingly, it can be deduced that the application of CTS-Arg NPs at 0.5% to plum fruit had an effective role in controlling chilling injury by increasing the content of proline.

### Phenolic compounds and PAL and PPO enzymes activity

**Fig. 6:**
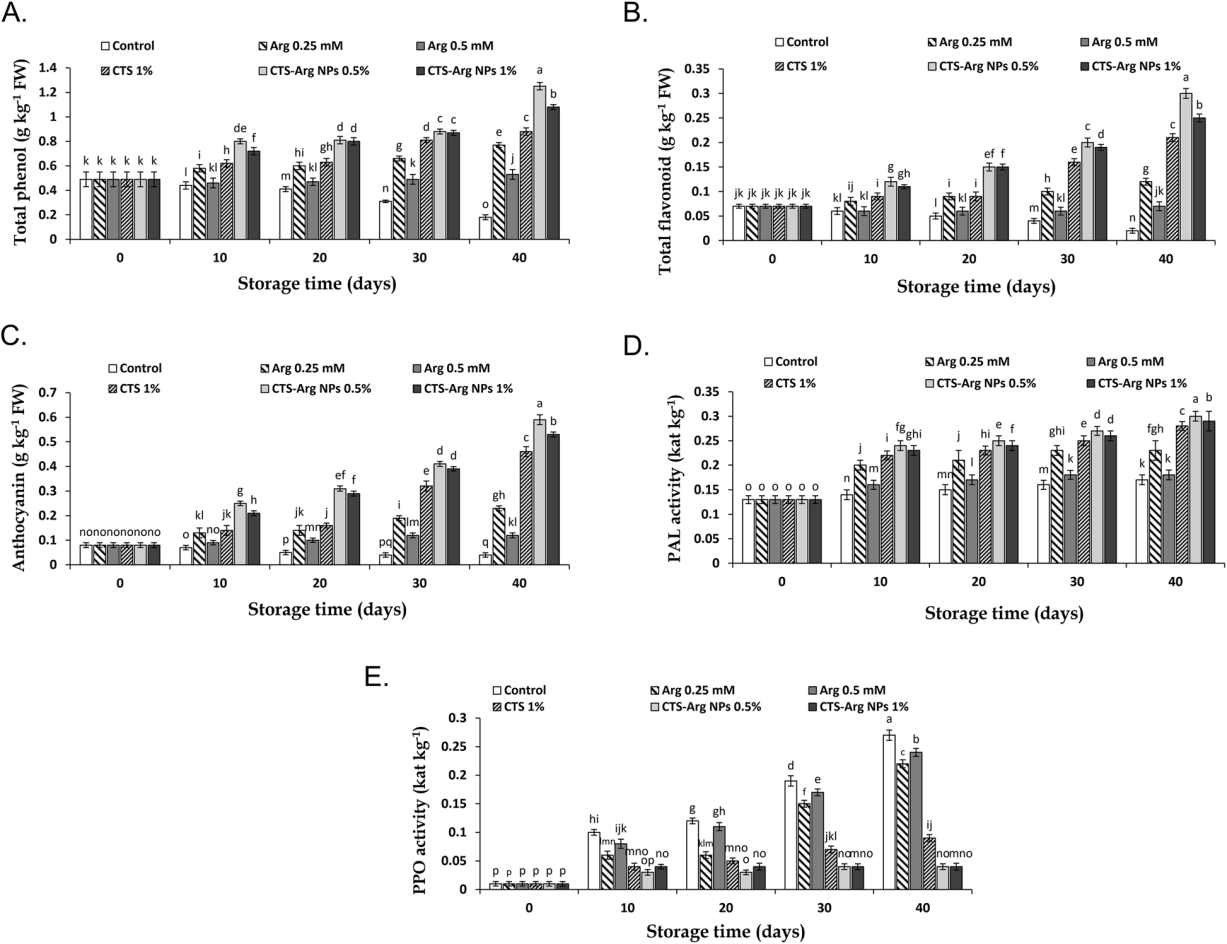
Effect of control (no treatment), arginine (Arg), chitosan (CTS) and CTS-Arg nanoparticles (NPs) treatments on total phenols (**A**), total flavonoids (**B**), anthocyanins (**C**), PAL activity (**D)** and PPO activity (**E**) in ‘Stanley’ plums during storage at 1 °C for 40 days. Values shown are means ± standard errors (*n* = 3). Different letters above the bars indicate significant differences at *P* < 0.05 according to Duncan’s test

Total phenol content, flavonoids, anthocyanins, PAL and PPO at harvest time are reported in Fig. 6. Treatments and storage time exhibited substantial efficacy on total phenols, flavonoids and anthocyanin content of plum fruit stored at 1 °C. Application of CTS-Arg NPs (0.5%) increased the content of phenols, flavonoids and anthocyanins in plums in comparison with control fruit. The amount of these compounds decreased in control samples during the entire cold-storage period (*P* < 0.01; Fig. 6A, B and C). Furthermore, plums coated with CTS-Arg NPs at 0.5% showed higher values of total phenols, flavonoids and anthocyanins (1.25, 0.3 and 0.59 g kg^− 1^ FW, respectively) than plums treated with CTS or CTS-Arg NPs (1%) during the 40-day storage period. In all samples, the activity of PAL enzyme increased during cold storage. However, this enhancement was more intensive in treated plums. At the end of the days storage period, the highest (0.3 kat kg^− 1^) and the lowest (0.17 kat kg^− 1^) values of PAL enzyme activity were observed in fruit treated with CTS-Arg NPs (0.5%) and control fruit, respectively (Fig. 6D). Furthermore, PAL activity enhanced in treated plums with 0.5 and 1% CTS-Arg NPs 0.3 and 0.29 kat kg^− 1^, respectively. The performance of 0.5% CTS-Arg NPs was continuously higher than 1% CTS-Arg NPs for increasing PAL activity. PPO enzyme activity increased during storage time in all treated and control fruit. At the end of the cold storage period, the activity of PPO in control fruit was higher than in fruit treated with CTS and CTS-Arg NPs at 0.5 and 1%. However, there were no differences among NPs treatments (Fig. 6E). PPO activity increased from its initial value of 0.01 kat kg^− 1^ to 0.27 kat kg^− 1^ in control fruits, while this value enhanced from 0.01 to 0.04 in fruits treated with CTS-Arg NPs after 40 days of cold storage.

Plums are a rich source of phenolic compounds, including phenols, flavonoids and anthocyanins, and the phenylpropanoid pathway is responsible for the biosynthesis and accumulation of these compounds in fruit [41, 42]. PAL is the main enzyme in the phenylpropanoid pathway and catalyzes the conversion of phenylalanine to trans-cinnamic acid, which plays an effective role in attenuating chilling injury [43, 44]. González–Aguilar et al. [45] reported that some edible coatings enhanced the accumulation of secondary metabolites such as phenolics, flavonoids and anthocyanins in tropical fruits. It was observed in the current study that fruit coating with CTS-Arg NPs (0.5%) increased the accumulation of phenolic compounds, which resulted from the higher activity of PAL enzyme. A similar effect was reported in grapes treated with an edible CTS-g-Salicylic acid coating [46]. Moreover, Babalar et al. [24] reported that chilling injury was attenuated in pomegranate fruit in response to Arg treatment, which induced an increment of phenolic compounds due to higher PAL and lower PPO enzymes activity. Our results are also in agreement with those by Xing et al. [27], who noticed that the application of a composite CTS-TiO_2_ coating maintained the postharvest quality of blueberry by increasing the content of phenolics, flavonoids and anthocyanins as well as preventing pigment demolition. Seemingly, CTS-Arg NPs treatment might activate the biosynthesis pathways of phenolic compounds and indirectly regulate the antioxidant defense systems in plum fruit. Flesh browning in stored fruits is an important problem that occurs due to oxidation and polymerization of phenolic compounds regulated by the activity of the PPO enzyme [47, 48]. The present findings revealed that unlike PAL, PPO enzyme activity had a negative effect on plum phenolic content. The activity of PPO enzyme was enhanced in control plums during the entire period of storage. Coating with CTS-Arg NPs maintained a lower level of PPO enzyme activity, in comparison to control fruit. Petriccione et al. [49] discussed that edible coatings could act as a semi-permeable barrier to oxygen responsible for polyphenol oxidase (PPO) reaction. Song et al. [28] reported that the oxidation of phenolic compounds during days of storage was attenuated in loquat fruit treated with CTS-silica, which was correlated with lower PPO activity in coated than in control fruit. The present findings suggest that CTS-Arg NPs coating induced resistance to chilling injury in plum fruit by decreasing the activity of PPO enzyme.

### Ascorbic acid content and DPPH scavenging capacity

**Fig. 7:**
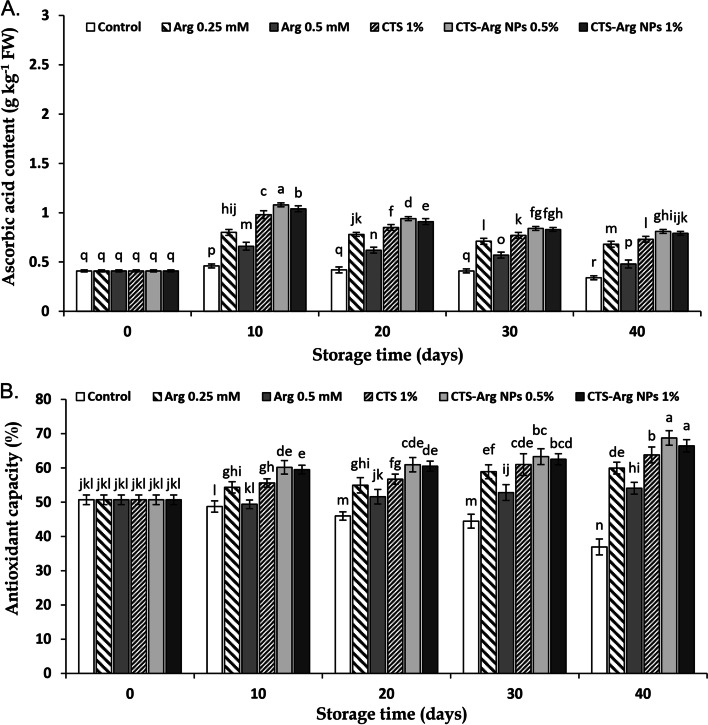
Effect of control (no treatment), arginine (Arg), chitosan (CTS) and CTS-Arg nanoparticles (NPs) treatments on ascorbic acid content (**A**) and antioxidant capacity (**B**) in ‘Stanley’ plums during storage at 1 °C for 40 days. Values shown are means ± standard errors (*n* = 3). Different letters above the bars indicate significant differences at *P* < 0.05 according to Duncan’s test

The amount of ascorbic acid of plum fruit at harvest was 0.41 g kg^− 1^ FW in this experiment, which deducted over time (Fig. 7A). During the first 10 days of storage, ascorbic acid content increased and then gradually decreased in both treated and control fruit during cold storage. However, plums coated with CTS-Arg NPs showed a higher accumulation of ascorbic acid than those treated with either CTS or Arg alone. There was no difference between CTS-Arg NPs at 0.5 and 1% concentration. As results in Fig. 7B showed the antioxidant capacity (DPPH scavenging activity) at harvest time was 50.69%. Plums coated with CTS-Arg NPs showed a higher DPPH radical scavenging activity than the rest of fruit during the storage period (Fig. 7B). CTS-Arg NPs at 0.5% were superior to 1% in increasing plum antioxidant capacity.

Ascorbic acid acts as (1) ROS scavenger and (2) an electron donor to APX for scavenging H_2_O_2_ by AA/GSH cycle [50, 51]. Based on the present results, ascorbic acid content was higher in plums treated with the CTS-Arg NPs coating than in control fruit. It might be thus deduced that higher DPPH scavenging activity in plum fruit coated with CTS-Arg NPs (0.5%) could result from higher content of phenolics, flavonoids, anthocyanins and ascorbic acid. In this sense, Sohail et al. [22] reported that postharvest Arg treatment prevented postharvest senescence of broccoli by reducing the ascorbic acid content and enhancing the antioxidant capacity. Likewise, the foliar application of CTS-phenylalanine nanocomposites at 5 mM concentration enhanced ascorbic acid content in grape fruit [52]. Babalar et al. [24] reported that the higher ascorbic acid accumulation in pomegranate fruit treated with arginine resulted from higher GR/APX system activity or lower AAO enzyme activity and remarkably assisted to increasing the antioxidant capacity of fruits during cold storage at 4 °C for 60 days. The use of chitosan nanoparticles loaded with *Citrus.aurantium* oil declined the loss of ascorbic acid content and increased the antioxidant capacity of white button mushroom during storage [53].

### Enzymatic antioxidant system activity and H_2_O_2_ content

**Fig. 8:**
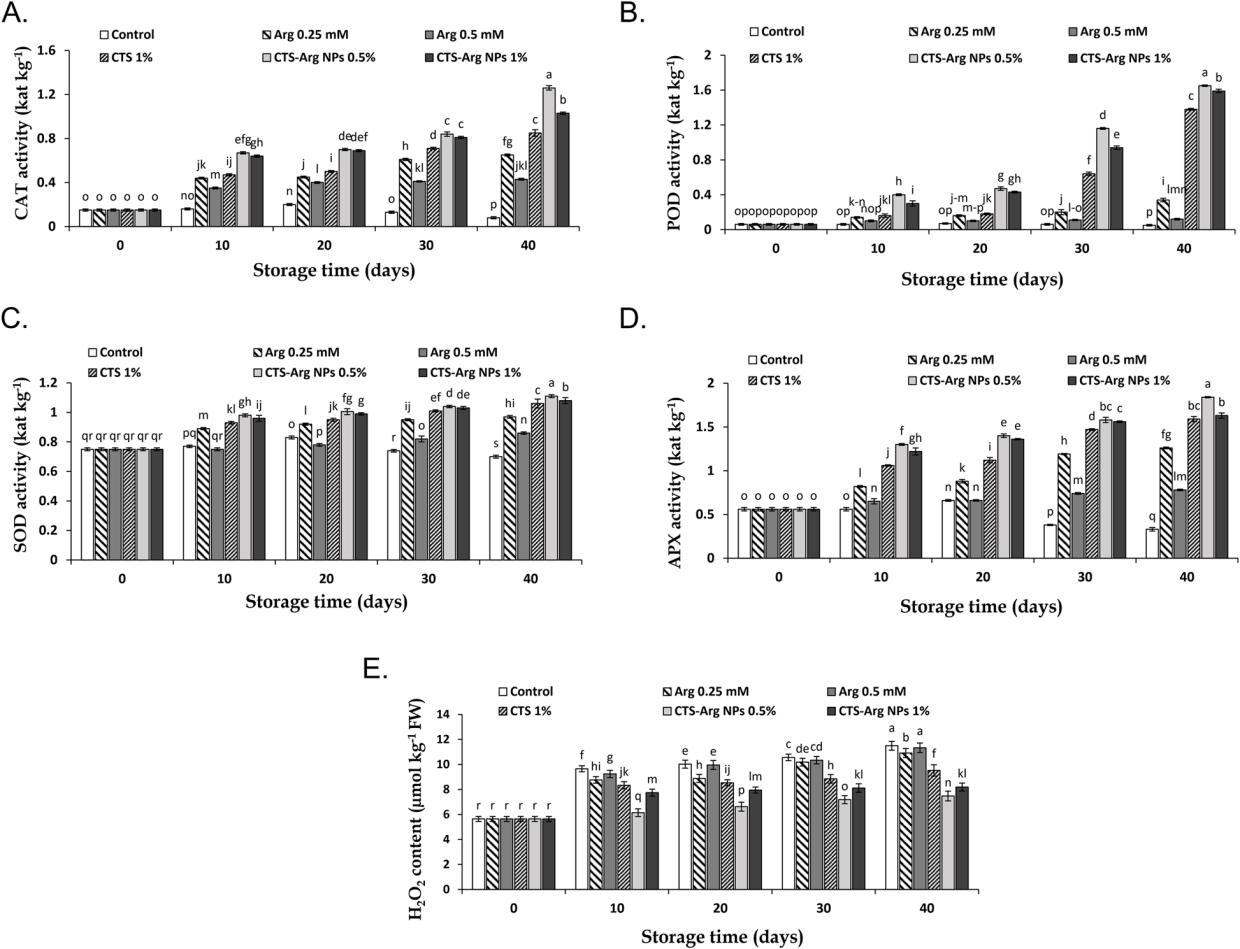
Effect of control (no treatment), arginine (Arg), chitosan (CTS) and CTS-Arg nanoparticles (NPs) treatments on catalase (**A**), peroxidase (**B**), superoxide dismutase (**C**) and ascorbate peroxidase (**D**) enzymes activity and H_2_O_2_ content (**E**) in ‘Stanley’ plums during storage at 1 °C for 40 days. Values shown are means ± standard errors (*n* = 3). Different letters above the bars indicate significant differences at *P* < 0.05 according to Duncan’s test

The activity of antioxidant enzymes and H_2_O_2_ content at harvest time are reported in Fig. 8. In the interval of 10 to 40 days of storage, the activity of antioxidant enzymes increased in all treated samples (Fig. 8A-D). Fruit coated with CTS-Arg NPs (0.5%) exhibited a higher activity of CAT, POD, APX and SOD than fruit treated with either CTS or Arg alone. Furthermore, control (no treatment) samples exhibited the highest enzyme activity up the first 20 days of storage and then gradually decreased. CAT activity enhanced in all treatments during cold storage. The activity of CAT enzyme was more stimulated than in control fruit after 40 days of storage. This enzyme activity in control fruit reduced after 20 days of storage (Fig. 8A). CTS-Arg NPs treatment increased the POD activity compared to the control fruit. There were significant different between CTS-Arg NPs and other treatments during the whole cold storage. In general, the concentration of 0.5% CTS-Arg NPs was the most effective treatment for increasing POD activity in plum fruits during 40 days of storage (Fig. 8B). Postharvest treatment of CTS-Arg NPs significantly increased the activity of SOD in plum fruits. The most activity of SOD was measured in treated plums with 0.5% CTS-Arg NPs (1.11 kat kg-1). The lowest SOD activity was found in control fruits after 20 days of storage (Fig. 8C). In all tested samples during 40 days of storage, APX activity enhanced in plum fruits. The highest APX activity was found in plums treated with CTS-Arg NPs in the storage period. The concentration of 0.5% CTS-Arg NPs was the most efficient treatment to increase the APX activity of plums during cold storage (Fig. 8D). As the storage period extended, the content of H_2_O_2_ increased in both treated and untreated fruit, although CTS-Arg NPs treatment alleviated the accumulation of H_2_O_2_ content (Fig. 8E). The highest and lowest values of H_2_O_2_ content were observed in control fruit (11.49 μmol kg^− 1^ FW) and fruit treated with CTS-Arg NPs at 0.5% (7.47 μmol kg^− 1^ FW), respectively. However, there was no substantial difference between control and 0.5 mM Arg treatments.

ROS are reduced forms of oxygen that cause oxidative stress under abiotic stress due to their producing and scavenging imbalance [54]. In order to scavenge accumulated ROS, two systems including enzymatic (CAT, POD, APX, SOD) and non-enzymatic (ascorbic acid) antioxidant systems are employed [29, 55]. SOD enzyme is responsible for scavenging ROS that converts superoxide (O_2_^•-^) radicals into H_2_O_2_ and O_2_^•^. Then, CAT catalyzes H_2_O_2_ into H_2_O and O_2_. Meanwhile, APX enzyme changes H_2_O_2_ into H_2_O through receiving an electron from ascorbate in the ascorbic acid/glutathione cycle [56, 57]. POD due to the oxidation of polyphenol compounds in presence of H_2_O_2_ is known in the browning process of fruits and vegetables [58]. Many studies showed that the increment of CAT, APX, SOD and POD activity was effective in controlling chilling injury and attenuating oxidative damage in plum fruit. Panahirad et al. [59] reported that pectin and carboxymethyl cellulose edible coatings reduced the accumulation of ROS due to the increases in POD activity. The application of L-cysteine after harvest reduced the browning of plum by increasing the activity of antioxidant enzymes (CAT, APX and SOD) and decreasing membrane degradation [60]. Our findings showed that plum coated with CTS-Arg NPs (0.5%) enhanced the activity of antioxidant enzymes during the storage period. Zhang et al. [61] stated that Arg treatment improved the CAT, POD and SOD activity, which increased disease resistance in tomato fruit. Likewise, in button mushrooms, Arg treatment inhibited cap browning and delayed senescence caused by undesirable environmental conditions through an increment of POD and SOD activity. In accordance with our results, previous studies found that the application of CTS NPs enhanced the activity of antioxidant enzymes in horticultural products such as loquat [28], plum [5] and tomato [62]. Shu et al. [19] reported that the antioxidant enzymes activities (CAT, POD, SOD, APX) enhanced after application of Arg 1 mM. They stated that the inducing effects of Arg on enzymatic antioxidant system activity may be one of its main mechanisms for preserving fruit quality. The present findings showed that the application of 0.5% CTS-Arg NPs remarkably decreased H_2_O_2_ content during cold storage. Babalar et al. [24] observed that pomegranate treated with Arg exhibited lower H_2_O_2_ content, resulting from the higher activity of antioxidant enzymes. Seemingly, the reduction of H_2_O_2_ content in CTS-Arg NPs-treated (0.5%) plums might be the consequence of the increased activity of antioxidant enzymes, as they are responsible for the elimination of H_2_O_2_.

### Heat map clustering and principal component analysis

**Fig. 9:**
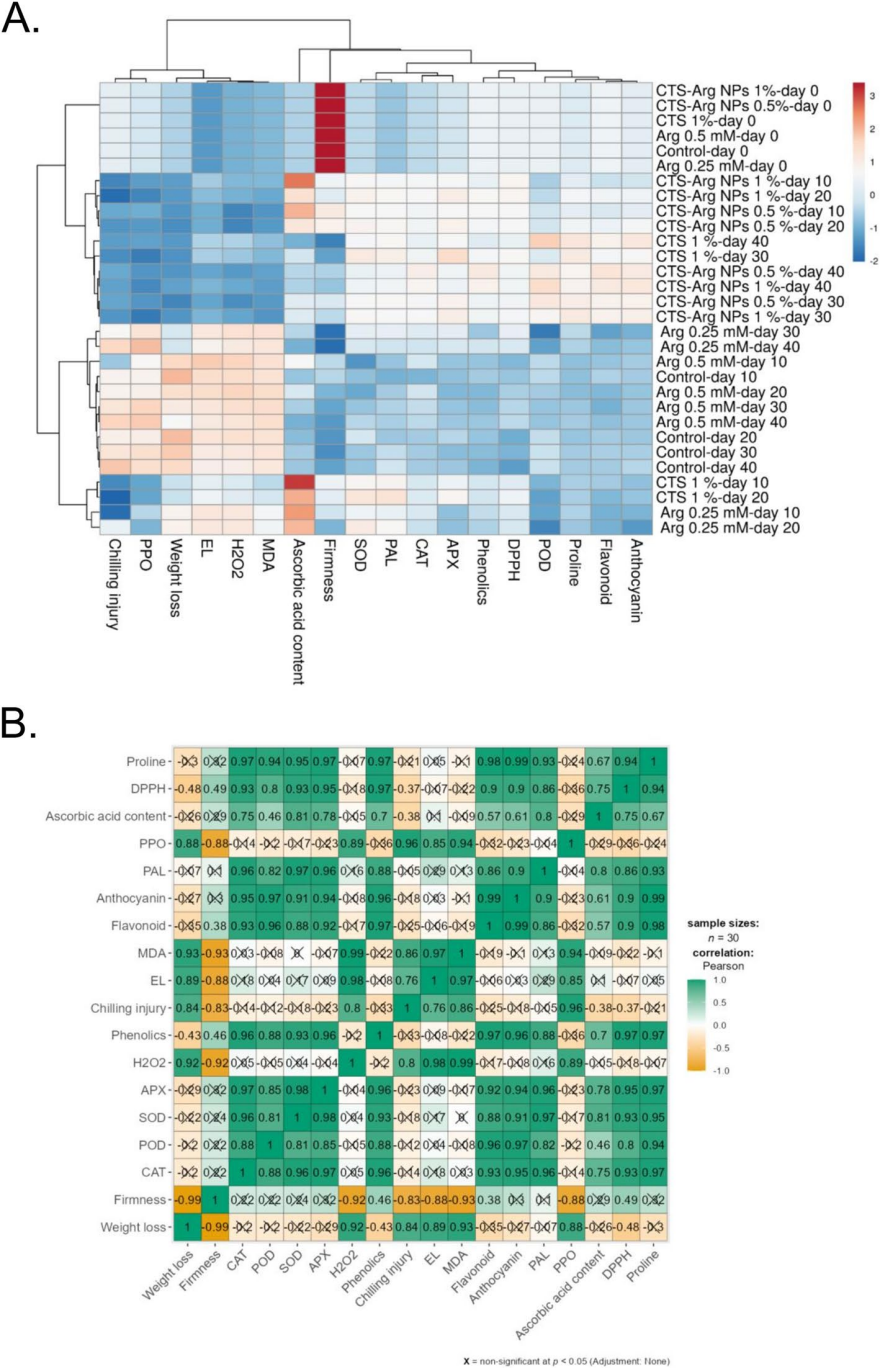
Heat map clustering (**A**) and correlation matrix of the assayed parameters (**B**) corresponding to plum quality parameters and application of postharvest treatments

Due to the high number of dependent and independent variables involved in this study, heat map clustering and principal component analysis (PCA) were used to analyze and visualize the results. According to the heat map clustering analysis, the findings are correlated and clear discrimination into two major clusters was observed for the treatments. The first cluster was mainly composed of CTS and its conjugation with Arg, with the exception of 1% CTS at day 10 and 1% CTS at day 20. The second cluster included the control and Arg groups. In the case of quality parameters, H_2_O_2_, MDA, EL, CI, PPO, weight loss, PAL, phenolic, flavonoid, and Ascorbic acid contents were sorted into the same cluster (Fig. 9 A, B).

**Fig. 10:**
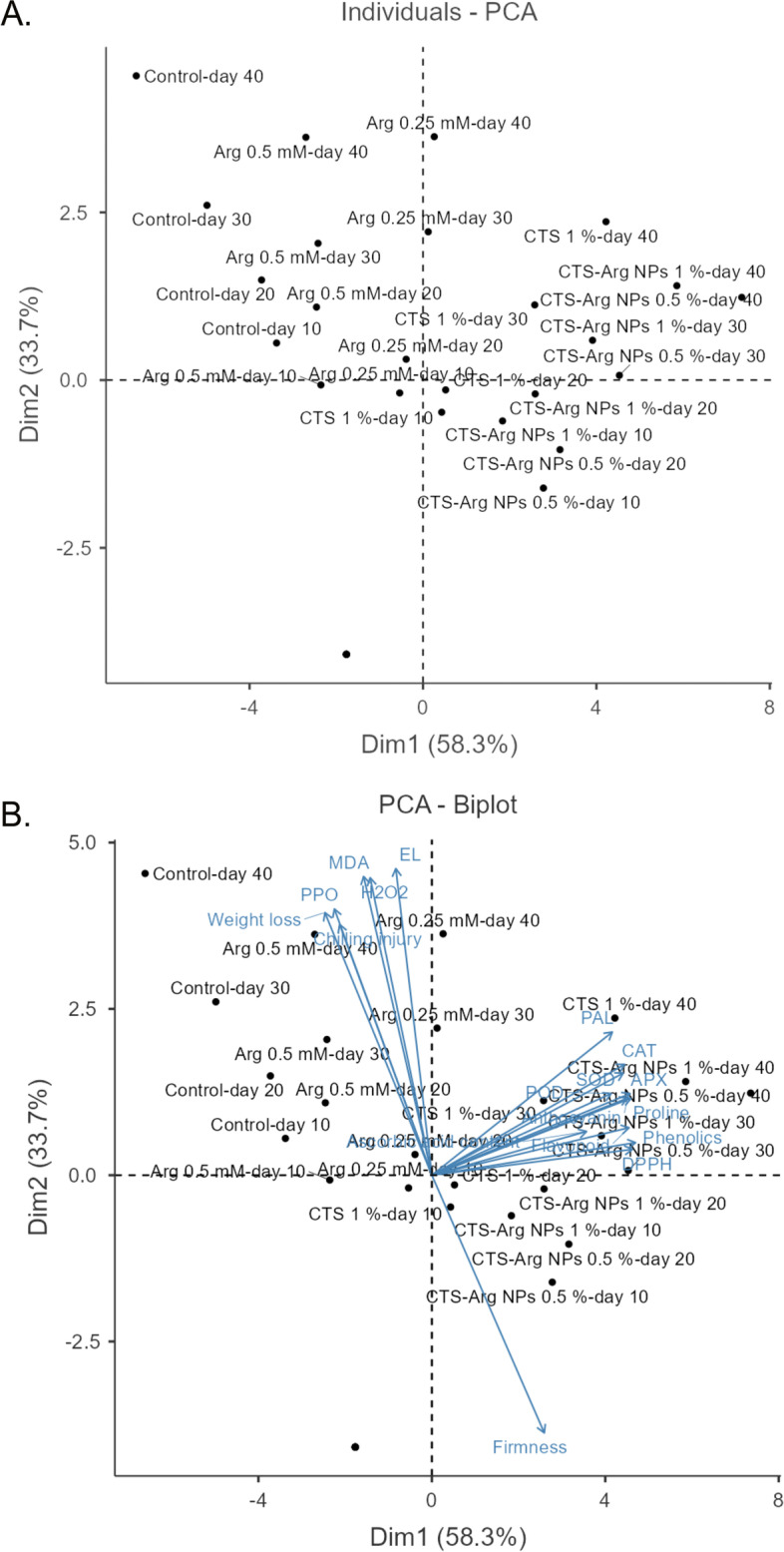
Principle component analysis (PCA) (**A**, **B**) corresponding to plum quality parameters and application of postharvest treatments

In general, relevant values were more pronounced for Arg and control groups, whilst they were lower for CTS and its conjugations. On the contrary, the higher values of antioxidant enzymes activity, firmness, anthocyanin and DPPH scavenging activity were observed for CTS and related groups. In addition, two PCA analyses with Eigenvalues > 1.0 (F1: 58.3%, F2: 33.7% accounting for 92.00% variability of the original data) showed that the first component (F1) had positive correlations with Ascorbic acid content, proline content, firmness, SOD, POD, PAL, CAT, APX, DPPH, phenolics, flavonoid and anthocyanin whereas the second component (F2) was positively correlated with H_2_O_2_, MDA, EL, chilling injury, PPO, and weight loss (Fig. 10A, B).

## Conclusion

The present results revealed that fruit coating with CTS-Arg NPs increased the chilling tolerance by reducing the accumulation of MDA and H_2_O_2_, increasing proline accumulation and ROS scavenging CAT, POD, APX and SOD enzymes activity and retaining the higher value of ascorbic acid in plum fruit stored at 1 °C and 90% RH for days. Moreover, the increment in PAL/PPO activities ratio lead to enhanced accumulation of phenolic compounds (phenols, flavonoids and anthocyanins), higher capacity of DPPH and reduced chilling injury in stored plums. Therefore, postharvest application of CTS-Arg NPs (especially at a concentration of 0.5%) showed potential as a harmless and safe treatment for retaining fruit quality and delaying ripening of plum fruit during cold storage.

## Materials and methods

### Chemical and plant material, treatments and storage

All chemicals including Chitosan (CTS) (Molecular Weight = 110 kDa and Deacetylation Degree = 84%, purity = 99%) sodium tripolyphosphate (TPP) (Molecular Weight = 367.847 g mol^− 1^), L-Arginine (Arg) (Molecular Weight = 174.20 g mol^− 1^) were purchased from Sigma - Aldrich, USA. Plum fruit (*Prunus domestica* cv. ‘Stanley’) at maturity stage were collected from a commercial orchard located in Khorramdareh (Zanjan Province, Iran) and transferred to the laboratory of postharvest physiology, University of Zanjan. Then, 504 plums were selected in terms of shape, size, colour, maturity and absence of visual damage. The fruits were harvested in commercial maturity stage and randomly assigned into 6 groups of 21 fruits for each treatment in triplicate (7 fruit per replicate) for 4 different storage time. The following treatments were applied as 10-min dips at 25 °C: control (distilled water), Arg at two concentrations (0.25 and 0.5 mM), CTS at 1% and Arg-CTS nanoparticles (CTS-Arg NPs) at two concentrations (0.5 and 1%, w/v). All nanomaterials were synthesized at laboratory of Nanochemsitry, University of Maragheh, Maragheh, Iran according to Nasr et al. [31] and Mahmoudi et al. [40]. A scanning electron microscope (SEM, VEGAII, XMU, Czech Republic) and transmission electron microscope (TEM; Philips CM10) was used to examine the surface morphology and the size of CTS-Arg NPs. Particle size and zeta potential for CTS-Arg NPs was recorded using a laser-scattering technique (DLS/Zeta; Zetasizer Nano ZS90; Malvern Instruments). Treated fruits were air-dried at room temperature and immediately stored at 1 °C and 90% relative humidity (RH) for 40 days. Sampling for assessment of physiological and biochemical characteristics was conducted at regular intervals of 10, 20, 30 and 40 days. For each evaluation time, prior to the performance of measurements and analyses, fruits were subjected to a shelf-life period of 2 day at 25 °C. Furthermore, fruit pulp was used to evaluate the biochemical attributes.

### Chilling injury, electrolyte leakage (EL) and malondialdehyde (MDA) content

Chilling injury (CI) index was assessed by estimating the area of the internal fruit tissue showing browning symptoms after 10, 20, 30, and 40 days of storage at 1 °C followed by 2 day of shelf life at 20 °C. Three independent replicates (*n* = 7 fruit each) were used for each treatment. Internal browning was computed as follows:

$$0=\textrm{no}\ \textrm{browning}\ \left(\textrm{good}\ \textrm{quality}\right);1=\textrm{slight}\ \textrm{browning};2=<1/4\ \textrm{browning};3=1/4-1/3\ \textrm{browning};4=1/3-1/2\ \textrm{browning}\ \textrm{and}\ 5=>1/2\ \textrm{browning}\ \left(\textrm{poor}\ \textrm{quality}\right)$$

The browning index (%) was calculated as in Aghdam et al. [63]:

$$\textrm{nternal}\ \textrm{browning}=\Sigma\ \left[\left(\textrm{browning}\ \textrm{level}\right)\times \left(\textrm{number}\ \textrm{of}\ \textrm{fruit}\ \textrm{at}\ \textrm{each}\ \textrm{browning}\ \textrm{level}\right)/\left(5\times \textrm{total}\ \textrm{number}\ \textrm{of}\ \textrm{fruit}\ \textrm{in}\ \textrm{the}\ \textrm{treatment}\right)\right]\times 100$$

The method of Promyou et al. [64] was used for determining the electrolyte leakage (EL):

$$\mathrm{EL}(\%)=(\mathrm{initial}\;\mathrm{EL}/\mathrm{final}\;\mathrm{EL})\times100$$

Determination of MDA content was carried out using the thiobarbituric acid (TBA) method proposed by Dhindsa et al. [65]. The content of MDA was expressed on a fresh weight basis, in μmol kg^− 1^ fresh weight (FW).

$$\textrm{MDA}=\left[\left(\textrm{A}532-\textrm{A}600\right)\times \textrm{W}\times \textrm{V}/155\right]\times 1000$$

(A: absorbance; W: weight samples; V: total volume of the aqueous extract)

### Weight loss and firmness fruit

The weight of fruit was measured using a digital balance at the beginning and end of each storage period and weight loss was calculated as the percentage of initial weight using the following formula [66]:

$$\textrm{Weight}\ \textrm{loss}\ \left(\%\right)=\left(\textrm{FW}\ \textrm{before}\ \textrm{storage}-\textrm{FW}\ \textrm{after}\ \textrm{storage}\right)/\textrm{FW}\ \textrm{before}\ \textrm{storage}\times 100$$

The firmness of plum fruit was analyzed using a texture analyzer (FT011), fitted with an 8-mm spherical probe and expressed as Newtons (N).

### Determination of proline content

The proline content was estimated according to the method described by Sánchez et al. [67]. Proline concentration was calculated using a standard curve constructed using proline and expressed as g kg^− 1^ FW.

### Total phenols, flavonoids, anthocyanin, and phenylalanine ammonia-lyase (PAL) and polyphenol oxidase (PPO) enzymes activity

The procedure of Folin–Ciocalteu was applied for evaluating total phenols content [68] They were expressed as gallic acid equivalents (GAE) on FW basis, g kg^− 1^. Total amount of flavonoids in the extracts was quantified according to the aluminum chloride colorimetric method, as described by Zhishen et al. [69]. The accumulation of flavonoids was expressed as quercetin equivalents (QE) on FW basis, g kg^− 1^. The pH-differential procedure described by Giusti and Wrolstad [70] was employed for determining total anthocyanin content, which was expressed as cyanidin-3-glucoside on FW basis, g kg^− 1^. The activities of PAL and PPO were quantified as described by Nguyen et al. [71] and expressed as katals produced per mass of protein, kat kg^− 1^.

### Ascorbic acid content and 2,2-diphenyl-1-picrylhydrazyl (DPPH) scavenging capacity

Total ascorbic acid content in plum fruit was evaluated using the 2,6-dichlorophenol indophenols method [72] and expressed on FW basis, g kg^− 1^. Free radical DPPH• scavenging activity was measured as described by Dehghan and Khoshkam [73] and calculated with the formula:

$$\%\,\text{DPPH}\cdot\text{inhibition}=\left(\text{Absorbance}\;\text{of}\;\text{control}-\text{Absorbance}\;\text{of}\;\text{sample}\right)/\text{Absorbance}\;\text{of}\;\text{control}\times100$$

### Determination of antioxidant system activity and hydrogen peroxide (H_2_O_2_) content

In order to reveal the activity of the enzymatic antioxidant system in the fruit samples, the activities of catalase (CAT), peroxidase (POD), superoxide dismutase (SOD), and ascorbate peroxidase (APX) were measured [74] and expressed as katals produced per mass of protein, kat kg^− 1^. Bradford [75] method was applied to determine protein content using bovine serum albumin (BSA) as a standard. H_2_O_2_ content was quantified according to the method of Alexieva et al. [76] and expressed in μmol kg^− 1^, considering a standard curve.

### Statistical analysis

The experiments were performed using a completely randomized factorial design. Each treatment was applied to three replications of seven fruit each. Analysis of variance (ANOVA) was carried out with SPSS software version 20 (SPSS Inc., Chicago, IL, USA). Differences between means were determined by Duncan’s test, with differences considered significant at *P* ≤ 0.05. For multivariate analysis; Clustvis and JAMOVI software were used.

## Data Availability

The datasets used and/or analysed during the current study are available from the corresponding author on reasonable request.
